# Research progress of quantitative electroencephalography in post-ischemic stroke mental disorders

**DOI:** 10.3389/fneur.2025.1445962

**Published:** 2025-04-15

**Authors:** Ai-ling Liu, Ming-hao Du, Yu-lei Liu, Cheng-jing Fei, Yu-qian Xue, Rong Yin

**Affiliations:** ^1^First School of Clinical Medicine, Gansu University of Chinese Medicine, Lanzhou, China; ^2^Neurology Department, Gansu Provincial Central Hospital, Lanzhou, China

**Keywords:** quantitative electroencephalography, ischemic stroke, mental disorder, prognostic indicators, time effects

## Abstract

Quantitative electroencephalography (qEEG) has significantly advanced in the field of neuroscience as a highly sensitive tool for routine monitoring following a stroke. It holds promise in diagnosing post-stroke psychiatric disorders and evaluating treatment outcomes. This review systematically examined published papers and thoroughly analyzed research findings on using qEEG indicators to monitor mental abnormalities in patients with ischemic stroke. The review covers key time periods, including the early stage (within 72 h), subacute stage (72 h to 1 month), and chronic stage (over 1 month) post-onset. The current evidence suggests that correlation indicators from electroencephalography (EEG) monitoring vary across different time periods, with Power spectrum analysis is a current research hotspot. This review summarizes and analyzes specialized studies on utilizing these qEEG indicators for monitoring and evaluating mental disorders in ischemic stroke, identifying key monitoring indicators highlighted in current research.

## 1 Introduction

As the trend of population aging intensifies and living standards continue to improve, the incidence of cerebrovascular diseases is on the rise (1). Stroke is categorized into ischemic and hemorrhagic types, with ischemic stroke being the most common, accounting for up to 87%. Despite advancements in science and technology, breakthroughs in stroke prevention, diagnosis, treatment, and rehabilitation have been significant, but the management of post-stroke complications remains challenging (2). Post-stroke mental disorders have garnered attention in the medical field, referring to emotional, cognitive, and behavioral abnormalities in stroke patients (3). The incidence is relatively high, posing obstacles to recovery and impacting quality of life (4). This issue has garnered the attention of various disciplines, including neurology, psychiatry, and rehabilitation medicine (5–8). Conducting in-depth multidisciplinary research on these disorders is essential for enhancing rehabilitation outcomes and improving patients’ quality of life.

The pathological process of stroke continues beyond the acute phase, triggering a series of long-term events. This includes alterations in cortical excitability, which can manifest early on and potentially precede clinical progression (9).

In clinical practice, the recovery and treatment of mental disorders following a stroke rely on a precise and continuous cycle of assessment, treatment, re-evaluation, and follow-up. Effective monitoring of post-stroke mental disorders has become essential, necessitating the establishment of a personalized assessment system to clarify the mental state post-stroke and develop tailored treatment and rehabilitation programs. Several research teams have conducted comparative analyses on various monitoring methods (5, 10–12) with the aim of providing patients with timely, effective, and more targeted interventions. Among these methods, high temporal resolution and non-invasive EEG have emerged as key tools for the rapid assessment of brain function, owing to their sensitivity to acute changes in cerebral blood flow and neuro metabolism (13). EEG analyzes the spontaneous and rhythmic electrical activity of cerebral cortical neuron groups, effectively reflecting brain functional status. However, conventional EEG’s high level of expertise and long reading time present challenges for immediate assessment by medical staff. In contrast, qEEG utilizes power spectrum analysis technology to convert EEG waveform amplitude into a frequency-related power display, presenting results in digital form, thereby objectively enhancing diagnostic accuracy and sensitivity, facilitating easier assessment by clinical staff. Research indicates that nearly 30% of patients with ischemic stroke will develop mood disorders, yet the trajectory of their development remains unclear. Identifying the time point and characteristic electrophysiological indicators when mood disorders are most likely to increase post-stroke is a critical aspect of stroke follow-up. Research indicates that stroke survivors and their families often express dissatisfaction with the information provided by healthcare providers, revealing a significant gap in their understanding of stroke and related issues (14–16). Identifying the time point and specific electrophysiological indicators at which mood disorders are most likely to escalate following a stroke is a crucial component of stroke follow-up. This understanding can enhance early intervention and support for patients and their families, addressing the associated risks effectively.

## 2 Methods

### 2.1 Review standards

To examine the current status and potential advancements of resting-state qEEG metrics in stroke, a systematic literature search was conducted. The review adhered to the Preferred Reporting Items for Systematic Reviews and Meta-Analyses (PRISMA) (17) guidelines. Articles were chosen based on specific research questions.

### 2.2 Research questions

We developed the following questions that guided our review.

Q1: Which qEEG features are associated with post-stroke mental disorders?

Q2: Are the changes in mental disorder-related parameters monitored by qEEG at different time periods consistent?

### 2.3 Search strategy

Quantitative EEG has seen increased usage in assessing mental disorders in ischemic stroke patients. However, factors like delays in treatment, referral processes, and varied examination plans have led to differences in monitoring time among research teams. A search on PubMed for relevant articles as of May 2024 with keywords like ‘quantitative electroencephalography’, ‘ischemic stroke’ and ‘mental disorders’ was conducted. The specific inclusion and exclusion criteria are summarized in the PICO criteria table (Table 1). The selected research papers were categorized into three groups based on the time from stroke onset to EEG monitoring initiation: (1) Within 72 h of onset; (2) Within 72 h to 1 month; (3) 1 to 3 months post-onsets. Those reporting multiple results were categorized across multiple time periods for detailed analysis.

**Table 1:** Characteristic qEEG indices in different time-periods.

	Patients with ischemic stroke and post-stroke mental disorders
Intervention	qEEG monitoring at various time points post-stroke onset (within 72 h, 72 h to 1 month, and over 1 month)
Comparison	Comparison of qEEG indicators at different time periods post-stroke onset
Outcome	Identification of specific qEEG features associated with post-stroke mental disorders
Study design	Any original study (cohorts, clinical trials, cross-sectional studies, case–control studies, and case series)
Exclude	1. Studies on patients with comorbid severe brain diseases (such as craniocerebral trauma, brain tumors, epilepsy, intracerebral hemorrhage, severe cognitive impairment, etc.), those who have been taking psychotropic drugs for a long time, and patients with severe underlying diseases (such as severe cardiorespiratory dysfunction, electrolyte disorders, hyperthyroidism, etc.). 2. Studies with an insufficient sample size (*n* < 20), studies with ambiguous reports of qEEG parameters, and studies with duplicate or overlapping data. 3. Studies using non-standardized diagnostic tools and studies lacking ethical approval.
Limits	English language only
Timespan	Published before May 2024

### 2.4 Paper selections

The initial search results were retrieved and uploaded onto Rayyan QCRI (18). Following the removal of any duplicate entries, two of the authors, identified as AL and MD, proceeded with an initial screening of the articles based solely on their titles and abstracts. This blind screening process consisted of the authors making decisions on each article’s inclusion, exclusion or uncertainty status. Once this initial screening phase is completed, the blind review mode will be discontinued, and any disagreements or conflicts that arise will be addressed and resolved by the corresponding author, RY. Subsequently, two of the researchers independently assessed the quality of each study using the Newcastle-Ottawa Scale (NOS) and conducted scoring and risk-of-bias assessments based on the scale. Any disagreements were resolved through discussion and consensus with RY. The assessment results show that three studies scored 7 points, three studies scored 8 points, and the remainder achieved full marks. Subsequently, we performed a bias analysis based on the NOS scores. Since all 22 studies scored 7 points or higher, they are all classified as having low bias.

### 2.5 Results

**Figure 1:**
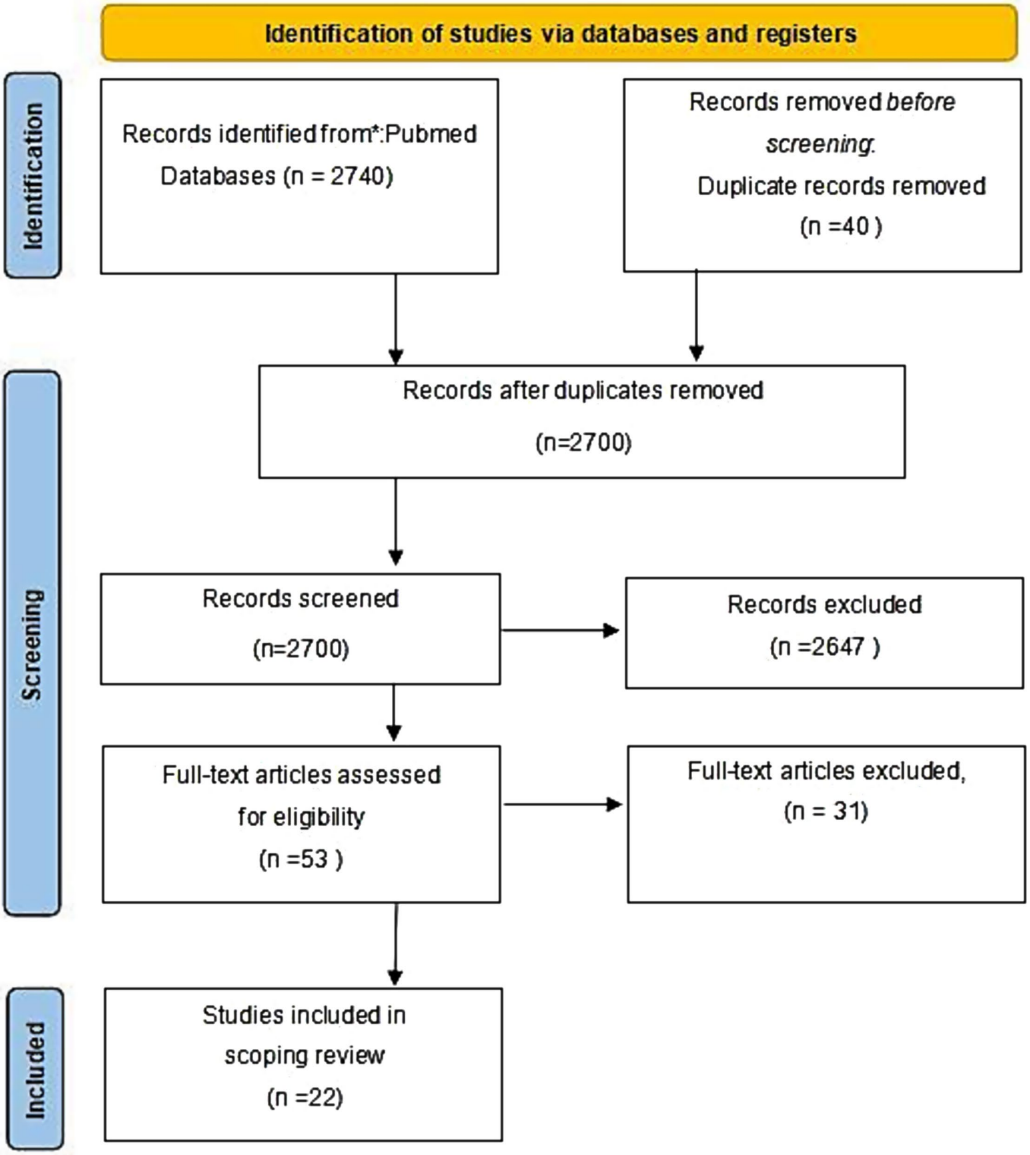
PRISMA flow diagram for systematic reviews.

A systematic search of relevant documents yielded 2,740 papers, which were then deduplicated to 2,700. Following initial screening based on abstracts and titles, 2,400 papers were excluded for not meeting the inclusion criteria, leaving 53 papers for further evaluation. In the process of thoroughly evaluating 53 papers, we identified that 31 of these papers did not meet our specified criteria for inclusion. Consequently, they were excluded from further consideration. After applying these exclusion criteria, we found that 22 papers fulfilled all the necessary conditions and were thus deemed eligible for inclusion in our review. The flow chart of literature selection is illustrated in Figure 1. For a detailed summary of each study’s characteristics, readers are directed to view Table 2 for detailed information.

**Table 2:** Population, intervention, comparison, outcome (PICO), inclusion and exclusion criteria.

Title	Year	First author	Time of EEG recording	Research types	Selection	Comparability	Outcome	NOS score	Bias risk	Research participants	Categories of mental disorders	Sample size	Monitoring tools	Research indicators	Conclusion
Associations Between EEG Beta Power Abnormality and Diagnosis in Cognitive Impairment Post Cerebral Infarcts	2012	Wang Y	Within 10 h	Cross-sectional study	4	2	3	9	Low	Cerebral infarct patients with cognitive impairment (CI-CI) and cognitive normality (CI-NC); Normal controls (NC)	Cognitive impairment post-cerebral infarcts	CI-CI: 65; CI-NC: 45; NC: 110	16-channel EEG (10–20 system)	Relative beta power; K-means clustering algorithm; MoCA scale	CI-CI patients have significantly lower EEG beta power compared to CI-NC and NC groups. Beta power is negatively correlated with infarct size and number. K-means clustering analysis shows good concordance with MoCA scoring for cognitive impairment assessment.
Acute single channel EEG predictors of cognitive function after stroke	2017	Anna Aminov	Within 72 h	Acute clinical study	4	2	2	8	Low	24 participants (23 completed the study)	Post-stroke cognitive function	23 (18 stroke patients, 5 controls)	Single-channel wireless EEG	Relative Power (RP) of delta, theta, alpha, beta, Delta/Alpha ratio (DAR), Delta/Theta ratio (DTR)	Acute DAR and DTR are potential predictors of post-stroke cognitive outcome.
Background Rhythm Frequency and Theta Power of Quantitative EEG Analysis: Predictive Biomarkers for Cognitive Impairment Post–Cerebral Infarcts	2014	Yang Song	Within 72 h	Prospective cohort study	4	2	3	9	Low	Cerebral infarct patients	Cognitive impairment post-cerebral infarcts	105 (95 after exclusions)	16-channel EEG (10–20 system)	Background rhythm frequency (BRF); Relative δ, θ, α, β band power; Kaplan–Meier method; Cox regression	Low BRF and high θ band power are predictive biomarkers for cognitive impairment post-cerebral infarcts, with hazard ratios of 14 and 5, respectively. These biomarkers may be valuable for early prediction of cognitive impairment.
Frontal EEG delta/alpha ratio and screening for post-stroke cognitive deficits: The power of four electrodes	2014	Emma Schleiger	Within 62–101 h	Prospective Study	4	2	3	9	Low	Patients with middle cerebral artery (MCA) ischemic stroke	Post-stroke Cognitive Deficits	Analyzed cases (*n* = 20)	EEG (19 electrodes, reduced to 4 frontal electrodes)	Cognitive outcomes (FIM–FAM), EEG indices (DAR, alpha power)	Frontal delta/alpha ratio (DAR) and global alpha power significantly correlate with post-stroke cognitive outcomes, suggesting potential for early screening using a reduced electrode montage.
Predictive power of abnormal electroencephalogram for post-cerebral infarction depression	2018	Yan-ping Z	Within 7 days	Cross-sectional observational study	4	2	3	9	Low	Patients with ischemic stroke from Shenzhen Second People’s Hospital, China	Post-cerebral infarction depression	321	Natus Nicolet digital video EEG recording system (19-channel)	EEG features (alpha, beta, theta, delta activity) and Hamilton Depression Rating Scale (HAMD)	Low-amplitude alpha activity and slow theta activity in EEG are independent predictors of post-cerebral infarction depression
Poststroke QEEG informs early prognostication of cognitive impairment	2016	Emma Schleiger	Within 10 days	Prospective cohort study	4	2	3	9	Low	Middle cerebral artery ischemic stroke patients	Cognitive impairment post-stroke	Stroke group: 35 (12 female, ages 18–87); Normal control: Not specified	19-channel EEG (10–20 system)	Relative power of delta, theta, alpha, beta bands; Delta: alpha ratio; Peak alpha frequency; Montreal Cognitive Assessment (MoCA)	Relative theta power (4–7.5 Hz) from three posterior electrodes over the stroke-affected hemisphere can predict cognitive impairment with 77.4% accuracy. QEEG measures could inform early prognostication of poststroke cognitive outcomes.
Machine learning-based prediction of post-stroke cognitive status using electroencephalography-derived brain network attributes	2023	Lee M	Within 2 weeks	Machine learning-based study	4	2	3	9	Low	87 patients with acute ischemic stroke	Post-stroke cognitive impairment (PSCI)	87	EEG-derived brain network attributes	Montreal Cognitive Assessment (MoCA)	EEG-based network properties can predict cognitive outcomes after ischemic stroke.
Quantitative EEG as a Biomarker in Evaluating Post-Stroke Depression	2023	Livia Livint, Popa	Over 1 months	Prospective study	4	2	2	8	Low	57 patients with post-stroke depression, 14 healthy controls	Post-stroke depression (PSD)	71 (57 PSD patients, 14 controls)	Quantitative EEG (qEEG)	(Delta + Theta)/(Alpha + Beta) Ratio (DTABR), Delta/Alpha Ratio (DAR)	QEEG parameters can facilitate the identification of PSD.
EEG spectral exponent as a synthetic index for the longitudina assessment of stroke recovery	2022	J. Lanzone	In 10 days&over 2 month	Longitudinal study	4	2	3	9	Low	18 patients with mild to moderate mono-hemispheric Middle Cerebral Artery (MCA) ischaemic stroke, 16 healthy controls	Stroke recovery	34 (18 stroke patients, 16 controls)	Quantitative EEG (qEEG)	Spectral Exponent (SE), Power Spectral Density (PSD)	SE is a reliable readout of neurophysiological alterations and clinical outcomes after ischemic cortical lesion.
Multi Modal Feature Extraction for Classification of Vascular Dementia in Post-Stroke Patients Based on EEG Signal	2023	Sugondo Hadiyoso	Over 3 month	Cross-sectional study	4	2	3	9	Low	Post-stroke patients with normal cognition, mild cognitive impairment (MCI), and vascular dementia	Normal cognition, MCI, Vascular dementia	Normal: 18; Post-stroke MCI: 19; Post-stroke dementia: 13	19-channel EEG (10–20 system)	Relative power, coherence, signal complexity; Support Vector Machine (SVM) and K-Nearest Neighbor (k-NN) classification	The proposed QEEG method achieved the highest classification accuracy of 96% using Gaussian SVM with a sensitivity of 95.6% and specificity of 97.9%. This method can be an additional criterion for diagnosing vascular dementia in post-stroke patients.
Persistence of the effects of Cerebrolysin on cognition and qEEG slowing in vascular dementia patients: Results of a 3-month extension study	2010	Dafin F. Muresanu	Over 6 months	Open-label Extension Study	4	2	3	9	Low	Patients with mild to moderate vascular dementia (VaD)	Vascular Dementia	10 mL Cerebrolysin (*n* = 13), 30 mL Cerebrolysin (*n* = 11), Placebo (*n* = 9)	EEG (qEEG analysis)	Cognitive performance (ADAS-cog+), EEG power ratio (PR)	Cerebrolysin’s beneficial effects on cognition and qEEG activity persist for at least 12 weeks after treatment cessation.
The value of quantitative EEG in differential diagnosis of Alzheimer’s disease and subcortical vascular dementia	2009	M. Gawel	Over 6 months	Cross-sectional study	4	2	3	9	Low	62 patients with Alzheimer’s disease (AD), 31 with SVD, 14 healthy controls	Alzheimer’s disease (AD), Subcortical vascular dementia (SVD)	107 (62 AD patients, 31 SVD patients, 14 controls)	Quantitative EEG (QEEG)	Alpha/slow wave power ratios, Mean wave frequency	QEEG parameters can differentiate between AD and SVD.
A pilot study to evaluate the effects of Cerebrolysin on cognition and qEEG in vascular dementia: Cognitive improvement correlates with qEEG acceleration	2008	Dafin F. Muresanu	Over 6 months	Pilot Study	4	2	3	9	Low	Patients with mild to moderate vascular dementia (VaD)	Vascular Dementia	10 mL Cerebrolysin (*n* = 16), 30 mL Cerebrolysin (*n* = 15), Placebo (*n* = 10)	EEG (qEEG analysis)	Cognitive performance (ADAS-cog), EEG power ratio (PR)	Cerebrolysin improves cognitive performance and reduces EEG slowing in VaD patients, with a positive correlation between cognitive changes and qEEG activity.
Does EEG (visual and quantitative) reflect mental impairment in subcortical vascular dementia	2007	M. Gawel	Over 6 months	Cross-sectional study	4	2	2	8	Low	31 patients with subcortical vascular dementia (SVD), 14 healthy controls	Subcortical vascular dementia (SVD)	45 (31 SVD patients, 14 controls)	Visual EEG, Quantitative EEG (QEEG)	Alpha/slow wave power ratios, Mean wave frequency	Only QEEG parameters are correlated with mental impairment in SVD.
EEG power changes are related to regional cerebral glucose metabolism in vascular dementia	1999	B. Szelies	Over 6 months	Cross-sectional study	4	2	3	9	Low	Patients with vascular dementia	Vascular dementia	28	19-channel EEG (10–20 system); FDG-PET	EEG power in delta, theta, alpha, and beta bands; Regional cerebral glucose metabolism	EEG power is linked to glucose metabolism in vascular dementia, with specific regional dependencies. Delta power is inversely related to metabolism, while alpha power is directly related to metabolism in the occipital lobe.
Slower EEG alpha generation, synchronization and “flow”—possible biomarkers of cognitive impairment and neuropathology of minor stroke	2017	Jelena Petrovic	Within 72 h & over 6 months	Observational Study	4	2	3	9	Low	Patients with minor right middle cerebral artery (MCA) ischemic strokes and healthy controls	Cognitive Impairment after Stroke	Stroke (*n* = 10), Controls (*n* = 11)	EEG (19-channel system)	EEG alpha frequency, synchronization, cognitive assessments (MoCA, MoCA-MIS)	Slower EEG alpha generation, increased synchronization, and altered ‘alpha flow’ are potential biomarkers of cognitive impairment onset and post-stroke re-organizational processes.
Electrophysiological changes in poststroke subjects with depressed mood: A quantitative EEG study	2018	Chunfang Wang	1 month & 6 month	Cross-sectional study	4	2	3	9	Low	35 poststroke depressed, 24 poststroke nondepressed, 35 healthy controls	Post-stroke depression	94 (35 depressed, 24 nondepressed, 35 controls)	Quantitative EEG (qEEG)	Spectral power in delta, theta, alpha, beta1, beta2 bands	Increased delta, theta, and beta2 power in PSD subjects, particularly in temporal regions.
Quantitative Electroencephalographic Correlates of Post-Stroke Depression	1998	Fred Ulam	WITHIN 3 months	Cross-sectional study	3	2	1	7	Low	21 stroke patients (CVA)	Post-Stroke Depression (PSD)	21	Cadwell Spectrum 32 Digital EEG	Interhemispheric power asymmetries in frontal regions; Correlation with BDI and GDS	Greater left frontal power asymmetry is associated with higher depressive symptoms in post-CVA patients.
Relationship Between Abnormalities in Resting-State Quantitative Electroencephalogram Patterns and Poststroke Depression	2021	Xinyuan Li	Not explicitly stated	Cross-sectional Study	4	2	3	9	Low	Patients with poststroke depression (PSD) and poststroke nondepressed (PSND) subjects	Poststroke Depression	PSD (*n* = 11), PSND (*n* = 23)	EEG (spectral power analysis)	Depression severity (HDRS), EEG power in different frequency bands	Absolute powers of alpha and theta bands significantly distinguish PSD from PSND, with alpha1 power positively associated with depression severity.
Quantitative EEG Markers in Mild Cognitive Impairment: Degenerative versus Vascular Brain Impairment	2012	D. V. Moretti	Not explicitly stated	Cross-sectional study	4	2	3	9	Low	99 subjects with MCI due to cerebrovascular damage, 79 with degenerative impairment	Mild cognitive impairment (MCI)	178 (99 vascular, 79 degenerative)	Quantitative EEG (QEEG)	Relative power in delta, theta, alpha1, alpha2, alpha3 bands	EEG markers can differentiate between vascular and degenerative MCI.
Quantified Electroencephalographic Correlates of Relative Frontalor Parietal Hypoperfusionin Dementia	1996	Daniel Pozzi	Not explicitly stated	Cross-sectional study	3	2	2	7	Low	26 patients with dementia, 10 normal controls	Dementia	36 (26 dementia patients, 10 controls)	Quantified EEG (qEEG)	Relative power in delta, theta, alpha, beta1, beta2 bands	Higher theta relative power in patients with parietal hypoperfusion.
Multifocal Infarct Dementia Treated by Cyclandelate and Monitored by Quantitative	1978	Dr. G. F. A. Harding	Not explicitly stated	Double-blind controlled study	3	2	2	7	Low	20 patients with cerebral arteriosclerosis	Arteriosclerotic dementia	20	Quantitative EEG (QEEG)	EEG frequency	EEG frequency decreased during placebo phase and improved with cyclandelate treatment.

This systematic review encompasses a diverse range of study designs, including cross-sectional studies, case–control studies, acute clinical studies, prospective cohort studies, machine learning-based studies, longitudinal studies, open-label extension studies, crossover studies, and double-blind controlled studies. The most prevalent study types were cross-sectional studies and prospective studies, accounting for 32 and 18.5%, respectively. The sample sizes of these studies ranged from a minimum of 10 participants to a maximum of 321 participants. In terms of the timing of EEG monitoring relative to stroke onset, the studies were categorized into four groups:

<72 h: 3 studies (13.6%) (19–21)

72 h to 1 month: 4 studies (18.2%) (22–25)

>1 month: 11 studies (50%) (11, 26–35)

Not explicitly stated: 4 studies (18.2%) (36–39)

Regarding EEG monitoring tools, four types were identified across the 22 studies: 16-channel EEG: 3 studies (13.6%), 19-channel EEG: 6 studies (27.3%), Wireless single-channel EEG: 1 study (4.5%), other tools: 12 studies (54.5%). Among the studies specifying EEG tools, 19-channel and 16-channel EEGs were the most widely used, accounting for 27.3 and 13.6%, respectively. QEEG was explicitly mentioned in 11 studies. Although not explicitly stated in the remaining studies, the EEG indices mentioned, such as relative power ratios, spectral entropy, Delta/Alpha Ratio (DAR), and brain symmetry indices, are all indicative of qEEG analysis. The EEG indices reported in the studies can be classified into four categories: relative power (*δ*, *θ*, *α*, *β*, and *γ*): 68%, Frequency band ratios: 14%, EEG network attributes (connectivity and complexity): 9%, other EEG indices (spectral indices, etc.): 9%. These findings highlight the diverse application of EEG and qEEG in stroke research, with a significant focus on relative power indices as a primary measure.

## 3 Quantitative EEG monitoring indicators related to mental abnormalities at various stages of stroke

### 3.1 Quantitative EEG monitoring within 72 h after stroke

By utilizing spectral analysis, coherence analysis, and other techniques, qEEG can effectively highlight ischemic areas in the brain and evaluate their impact on the conduction of electrical signals. Research by Cuspineda et al. (40) emphasized the critical importance of the first 72 h post-stroke for qEEG monitoring, as electrophysiological examinations during this period are closely linked to patient recovery. The accurate diagnosis and selection of appropriate treatment for patients with mental disorders are greatly influenced by the complex and vague symptoms (41), the expertise of specialists, and the patient’s own account. This challenge is further compounded when patients also have physical disabilities resulting from stroke. Researchers are delving into objective biomarkers, such as qEEG, to gain insight into the mental status of these patients. Previous studies have revealed strong associations between specific qEEG parameters, such as frequency bands, power ratios, and symmetry index, and stroke severity as measured by the National Institute of Health Stroke Scale (NIHSS), as well as functional status as assessed by the modified Rankin Scale (42). Song et al. (19) examined the relationship between changes in qEEG frequency bands during the acute phase and changes in cognitive function three months post-stroke, revealing that certain early qEEG parameters could serve as predictive biomarkers of cognitive dysfunction. Schalk et al. (21) further expanded the role of qEEG in acute stroke monitoring by showcasing the relative band power of the delta, theta and alpha frequencies, as well as the importance of the power ratio DAR and delta/theta ratio (DTR) in acute stroke monitoring. And through regression analysis, it was determined that the most significant acute electrophysiological marker of long-term cognitive function was DTR. Studies have also been conducted on patients experiencing post-stroke psychosis, with the DTR demonstrating the highest predictive performance among the EEG parameters identified thus far. Subsequent research teams have conducted detailed analyses on more distinctive EEG parameters and specific brain regions.

Several studies have emphasized the clinical importance of frontal region alpha waves in evaluating cognitive impairment post-stroke (21, 32). Additionally, some researchers have also focused their attention on the theta band (20). Conversely, Öksüz et al. (43) study on central nervous system disorders revealed a notable link between decreased absolute power of alpha waves in the resting occipital lobe and cognitive impairment. EEG assessments within 72 h of stroke onset may exhibit some instability in mapping and potentially trigger abnormal electrical activity throughout the cerebral cortex. Consequently, further investigation is warranted to elucidate the connection between abnormal frequency alterations in different brain regions during the early stages of stroke and subsequent mental irregularities. Recent advancements in gamma-band research have underscored the pivotal role of gamma-band oscillations in sensory and cognitive functions. While past research has primarily focused on the association between high-frequency bands and cognition (44), the precise clinical implications of gamma bands remain ambiguous. A 2018 study examining resting-state gamma-band activity observed reduced gamma-band frequencies in individuals with depression and identified a strong correlation between changes in gamma power at the Cz electrode and the severity of mental disorders (45). However, studies on the gamma-band after ischemic stroke are relatively few, and the included studies did not involve this.

### 3.2 Quantitative EEG monitoring from 72 h to 1 month after stroke

A significant relationship was observed between qEEG monitoring and prognostic prediction within 72 h to 1 month of stroke onset, a critical period for early intervention and rehabilitation of acute stroke. Recent studies have demonstrated the potential of quantitative electroencephalography (qEEG) as a valuable tool for predicting and monitoring post-stroke cognitive impairment within the critical period of 72 h to 1 month after stroke onset. Giannakopoulos et al. (46) demonstrated that EEG can distinguish subtle differences between structural changes and metabolic defects in patients with progressive cognitive decline. Schleiger et al. (25) conducted a prospective study on patients with middle cerebral artery (MCA) ischemic stroke, recording EEG within 62–101 h post-stroke. They found that the frontal DAR and global alpha power significantly correlated with cognitive outcomes, suggesting that a reduced electrode montage could be used for early screening of cognitive deficits. Within a similar timeframe, Zheng et al. (23) identified low-amplitude alpha activity and slow theta activity in EEG recordings within 7 days of stroke onset as independent predictors of post-cerebral infarction depression (PCID) in 321 ischemic stroke patients. Schleiger et al. (24) further extended this research by demonstrating that relative theta power measured within 10 days post-stroke could predict cognitive impairment with 77.4% accuracy. More recently, Lee et al. (22) utilized machine learning to analyze EEG-derived brain network attributes within 2 weeks of stroke onset, showing that these properties could predict cognitive outcomes after ischemic stroke. Collectively, these studies highlight the value of qEEG in the early detection and prediction of cognitive and emotional complications following stroke, providing a basis for timely intervention and management strategies.

### 3.3 Quantitative EEG monitoring more than 1 month after stroke

The application of qEEG technology holds increasing value in patients with psychotic disorders in the later stages of stroke. Particularly when monitored one month after the onset of symptoms, qEEG results can assist doctors in more accurately assessing the condition of patients with mental disorders. Sheorajpanday et al. (42) highlighted the predictive value of delta and theta wave rhythms observed through qEEG one month post-stroke in determining patients’ functional recovery. They noted that changes in slow wave activity, especially in the affected hemisphere, are closely linked to prognosis in stroke patients. Popa et al. (29) conducted a prospective study on post-stroke depression (PSD), recording EEG over 1 month post-stroke and identifying specific qEEG parameters, such as the (Delta + Theta)/(Alpha + Beta) Ratio (DTABR) and DAR, that facilitate the identification of PSD. Lanzone et al. (28) further explored the longitudinal assessment of stroke recovery using the EEG spectral exponent (SE) as a synthetic index, demonstrating its reliability in reflecting neurophysiological alterations and clinical outcomes over 2 months. Hadiyoso et al. (11) extended this research by using EEG-derived brain network attributes to classify post-stroke patients with vascular dementia, achieving high accuracy with Gaussian SVM. Muresanu et al. (31) highlighted the persistence of cognitive benefits and qEEG improvements in patients with vascular dementia treated with Cerebrolysin over 6 months. Gawel et al. (27) differentiated Alzheimer’s disease from subcortical vascular dementia using qEEG parameters over 6 months, emphasizing the diagnostic value of EEG in degenerative conditions. Muresanu et al. (30) also demonstrated the correlation between cognitive improvement and qEEG acceleration in patients with vascular dementia treated with Cerebrolysin over 6 months. Gawel et al. (26) showed that only qEEG parameters were correlated with mental impairment in subcortical vascular dementia over 6 months. Szelies et al. (33) linked EEG power changes to regional cerebral glucose metabolism in vascular dementia, revealing specific regional dependencies. Petrovic et al. (32) observed slower EEG alpha generation and synchronization as potential biomarkers of cognitive impairment and neuropathology in minor stroke over 6 months. Wang et al. (35) identified increased delta, theta, and beta2 power in PSD subjects over 6 months, particularly in temporal regions.

Collectively, these studies underscore the importance of qEEG in the long-term monitoring and management of stroke patients. qEEG parameters not only provide valuable insights into cognitive and emotional outcomes but also offer potential biomarkers for early intervention and personalized treatment strategies. The consistent use of qEEG across various studies highlights its reliability and versatility in assessing long-term neurophysiological changes and guiding clinical decisions. Future research should focus on integrating qEEG with other diagnostic tools to enhance the accuracy and timeliness of interventions for stroke patients.

### 3.4 Quantitative EEG segmental monitoring after stroke

During the management of ischemic stroke, qEEG monitoring at various time points can provide valuable insights into the patient’s condition. Multiple qEEG monitoring sessions are particularly beneficial in assessing mental disorders, offering detailed information for clinical decision-making. Research indicates that changes in qEEG readings at different stages are closely linked to neurological functional recovery (42). Segmented qEEG monitoring aids in predicting the severity of mental disorders and potential recovery outcomes (21). Continuous qEEG monitoring is essential for mid-term to long-term follow-up, enabling doctors to detect any recurrence or complications early on and adjust treatment plans accordingly (47). Longitudinal qEEG monitoring can also shed light on the progression of psychiatric disorders post-cerebrovascular disease. For instance, Jelena et al. (32) conducted a study involving EEG evaluation within 72 h and 6 months post-stroke, revealing increased alpha synchronization in the ipsilateral hemisphere of the lesion during coherence analysis. This phenomenon was attributed to compensatory mechanisms around the lesion and in the affected brain region during the recovery phase following stroke, suggesting a potential correlation between inter-hemispheric alpha synchronization and cognitive recovery post-stroke. The article highlights the significant correlation between slow wave alpha and cognitive impairment across acute and chronic stages. In a recent study by Lanzone et al. (28), new monitoring indicators and a spectral index were introduced to reflect changes in power spectral density. Longitudinal assessment during subacute and chronic phases revealed a re-normalization of EEG signals over time, corresponding to partial cognitive recovery post-stroke. Correlation analysis demonstrated the potential of the spectral index as a specific monitoring indicator for post-stroke mental disorders.

## 4 Discussion

When delving into the relationship between qEEG and post-ischemic stroke mental disorders, this review study has found that multiple qEEG features are associated with such disorders, and the relevant parameters monitored at different time periods vary (for the specific characteristic electroencephalogram indices in each time period, please refer to Table 3). These two key issues are of great significance for understanding the research progress and future directions in this field. The following is a discussion.

**Table 3:** Characteristics of the included studies.

Time point	EEG index	Example data (comparison with baseline or control group)	Reference
<72 h	The decrease in Beta power	Beta Power: 0.832 ± 0.203 (Control: 1.565 ± 0.345), t(3.51) = −3.51, *p* < 0.01, d = 2.42	Wang et al. (11)
	Specific frequency bands, power ratios	DAR: 2.57 ± 1.88 (Control: 1.26 ± 0.59), t(21.46) = −2.88, *p* < 0.01, d = 0.93 RP delta: 0.39 ± 0.13 (Control: 0.28 ± 0.07), t(26.65) = 3.28, *p* < 0.01, d = 1.07 DTR: 1.60 ± 1.11 (Control: 0.87 ± 0.21), t(19.29) = −2.83, *p* = 0.01, d = 0.92 RP theta: 0.28 ± 0.06 (Control: 0.33 ± 0.05), t (36) = −2.56, *p* = 0.02, d = 0.83 RP alpha: 0.20 ± 0.08 (Control: 0.25 ± 0.07), t (36) = −2.02, *p* = 0.05, d = 0.66 RP beta: 0.11 ± 0.04 (Control: 0.13 ± 0.04), t (36) = 1.33, *p* = 0.44, d = 0.50	Anna Aminov (21)
	Low BRF and high Theta power	BRF < 7.4 Hz: 68% (Control: 9%), HR = 14, *p* < 0.001 Theta Power > 22%: 57% (Control: 26%), HR = 5, *p* = 0.003	Yang Song (19)
72 h-1 month	Frontal DAR and global Alpha power	Frontal DAR: ρ = −0.664, *p* ≤ 0.001 Global Alpha Power: ρ = 0.67, *p* ≤ 0.001	Schleiger et al. (25)
	increased of Low-amplitude alpha activity and slow theta activity	Alpha Activity: 1.85 ± 0.55 (Control: 1.00 ± 0.10), OR = 1.85, *p* = 0.017 Theta Activity: 1.76 ± 0.45 (Control: 1.00 ± 0.10), OR = 1.76, *p* = 0.033	Yan-ping (23)
	The relative power ratio of theta in the ipsilateral occipital lobe of the lesion	Occipital relative theta power greater than 0.150 was identified as the optimal criterion value to indicate cognitive impairment or lack thereof (Sensitivity: 0.800, Specificity: 0.687)	Schleiger et al. (24)
	Theta-band related characteristics	Global efficiency (Theta/ROI8): Correlation coefficient = 0.64	Lee et al. (22)
>1 month	Global DTABR and frontal extended DTABR	Global DTABR:1.516 ± 0.06 (Control: 1.440 ± 0.06), r = 0.292, *p* = 0.028 Frontal Extended DTABR:1.486 ± 0.05 (Control: 1.418 ± 0.05), r = −0.392, *p* = 0.003	Livia Livint, Popa (29)
	The decrease of Spectral Exponent.	SE (HC):-1.12 ± 0.24 SE (Stroke Patients at T0):-1.48 ± 0.27 (Control: −1.12 ± 0.24), t (19) = −3.24, *p* = 0.0029 SE (Stroke Patients at T1):-1.33 ± 0.30 (Control: −1.12 ± 0.24), t (19) = −1.54, *p* = 0.05	Lanzone et al. (28)
	Relative Power, Coherence and Complexity	Delta Band: 0.6664 ± 0.0036 (Control: 0.1936 ± 0.0036), *p* < 0.001 Theta Band: 0.595 ± 0.0036 (Control: 0.9075 ± 0.0036), *p* < 0.001 Alpha Band: 0.9407 ± 0.0036 (Control: 0.3107 ± 0.0036), *p* < 0.001 Beta Band: 0.013 ± 0.0036 (Control: 0.018 ± 0.0036), *p* < 0.001 Interhemispheric Coherence: 0.956 ± 0.001 (Control: 0.979 ± 0.001), *p* < 0.001 Spectral Entropy: 0.731 ± 0.001 (Control: 0.8 ± 0.001), *p* < 0.001	Hadiyoso et al. (11)
	The slow-to-fast power ratio	The slow-to-fast power ratio: 1.62 ± 0.12 (*N* = 9)	Muresanu et al. (31)
	Alpha/Theta and Alpha/Delta	Alpha/Theta: 0.75 ± 0.45 (Control: 4.60 ± 2.01) Alpha/Delta: 2.51 ± 2.19 (Control: 9.40 ± 2.72)	Gawel et al. (27)
	Relative Power	Theta Relative Power: 23.30 ± 1.82 Delta Relative Power: 34.80 ± 2.50 Alpha Relative Power: 27.26 ± 2.82 Beta Relative Power: 14.63 ± 1.00	Muresanu et al. (30)
	Alpha/Theta, Alpha/Delta and Alpha/(Theta + Delta)	Alpha/Theta SVD I: 2.25 ± 0.96 (Control: 4.60 ± 2.01), *p* < 0.05 Alpha/Theta SVD II: 1.28 ± 1.18 (Control: 4.60 ± 2.01), *p* < 0.05 Alpha/Delta SVD I: 3.60 ± 1.47 (Control: 9.40 ± 2.72), *p* < 0.05 Alpha/Delta SVD II: 2.51 ± 2.19 (Control: 9.40 ± 2.72), *p* < 0.05 Alpha/(Theta + Delta) SVD I: 1.23 ± 0.47 (Control: 2.84 ± 1.26), *p* < 0.05 Alpha/(Theta + Delta) SVD II: 0.77 ± 0.70 (Control: 2.84 ± 1.26), *p* < 0.05	Gawel et al. (26)
	The power of delta, alpha and theta waves	The power of delta and theta waves is significantly negatively correlated with local cerebral glucose metabolism (P < 0.01). The power of alpha waves is significantly positively correlated with local cerebral glucose metabolism (*p* < 0.01)	Szelies et al. (33)
	Frontal lobe alpha power, Slow-wave activity in the affected hemisphere	αAVG: 10.19 ± 0.19 (Control: 10.52 ± 0.22), χ2 ≥ 6.11; *p* ≤ 0.05;z ≥ −3.32; *p* ≤ 0.03 Increased theta and beta synchronizations at the F3–F4 electrodes (χ2 ≥ 7.67; *p* ≤ 0.02; z ≥ −2.84; *p* ≤ 0.02)	Petrovic et al. (32)
	Theta and Beta2 power in the right temporal area	Theta PSD: 1.50 ± 0.45 (PSND: 1.15 ± 0.35, HC: 1.05 ± 0.30) Beta2 PSD: 1.35 ± 0.50 (PSND: 1.10 ± 0.40, HC: 1.00 ± 0.35)	Wang et al. (35)
	Power Asymmetry	FP1-2 Power Asymmetry and BDI Scores: 0.68 ± 0.10, r = 0.68, *p* < 0.05 F3-4 Power Asymmetry and BDI Scores: 0.65 ± 0.10, r = 0.65, *p* < 0.05 FP1-2 Power Asymmetry and GDS Scores: 0.68 ± 0.10, r = 0.68, *p* < 0.05 F3-4 Power Asymmetry and GDS Scores: 0.65 ± 0.10, r = 0.65, *p* < 0.05	Ulam et al. (34)
Not explicitly stated	Differences in alpha power activity and the ratio of alpha to slow waves	Higher Alpha1 power in left temporal region (*p* = 0.019) The Alpha2 band was higher at left frontal pole (*p* = 0.031) Higher Theta power in central (*p* = 0.045), temporal (*p* = 0.049), and occipital (*p* = 0.026) regions	Li et al (37)
	Delta Band, Alpha2 Band, Theta/Alpha1 and Alpha2/Alpha3	Delta Band: 1.20 ± 0.35 (Control: 0.95 ± 0.25), t (57) = 2.34, *p* = 0.02, d = 0.61 Alpha2 Band: 0.85 ± 0.20 (Control: 1.10 ± 0.30), t (57) = 4.20, *p* < 0.001, d = 1.05 Theta/Alpha1: 1.39 ± 0.14 (Control: 0.70 ± 0.05), t (57) = 4.50, *p* < 0.001, d = 1.10 Alpha2/Alpha3: 0.79 ± 0.07 (Control: 1.27 ± 0.12), t (57) = 5.20, *p* < 0.001, d = 1.25	Moretti et al. (38)
	Relative Power	Theta Relative Power: 1.20 ± 0.30 (Control: 0.90 ± 0.20), t(16.29) = 3.50, *p* = 0.001, d = 0.92 Delta Relative Power: 1.10 ± 0.25 (Control: 0.85 ± 0.20), t(16.29) = 2.30, *p* = 0.02, d = 0.61 Alpha/Theta: 0.70 ± 0.15 (Control: 0.85 ± 0.10), t(16.29) = −2.80, *p* = 0.006, d = 0.78	Pozzi et al. (39)
	The rate of change of EEG frequency	The rate of change of EEG frequency: −0.0196 Hz / month ±0.003	Harding et al. (36)

Q1: Which qEEG features are associated with post-stroke mental disorders?

As can be seen from Table 3 and related studies, multiple qEEG features play crucial roles in the monitoring and assessment of post-stroke mental disorders. Within 72 h after stroke onset, specific frequency bands, power ratios (such as DAR and DTR), symmetry indices, frontal lobe alpha waves, and gamma-band oscillations are related to mental disorders. Among them, frontal lobe alpha waves are of great significance in the assessment of early-stage cognitive impairment (11, 32), and although the clinical significance of gamma-band oscillations remains unclear, they provide a new perspective for exploring the pathogenesis of mental disorders (44, 45, 48). Additionally, changes in the absolute power of occipital alpha waves have been associated with cognitive disorders (43). However, the specific mechanisms by which these features contribute to post-stroke mental disorders still need in-depth research. In the future, more basic and clinical studies are required to analyze their internal relationships and provide support for precise diagnosis and treatment.

Q2: Are the changes in mental disorder-related parameters monitored by qEEG at different time periods consistent?

**Figure 2:**
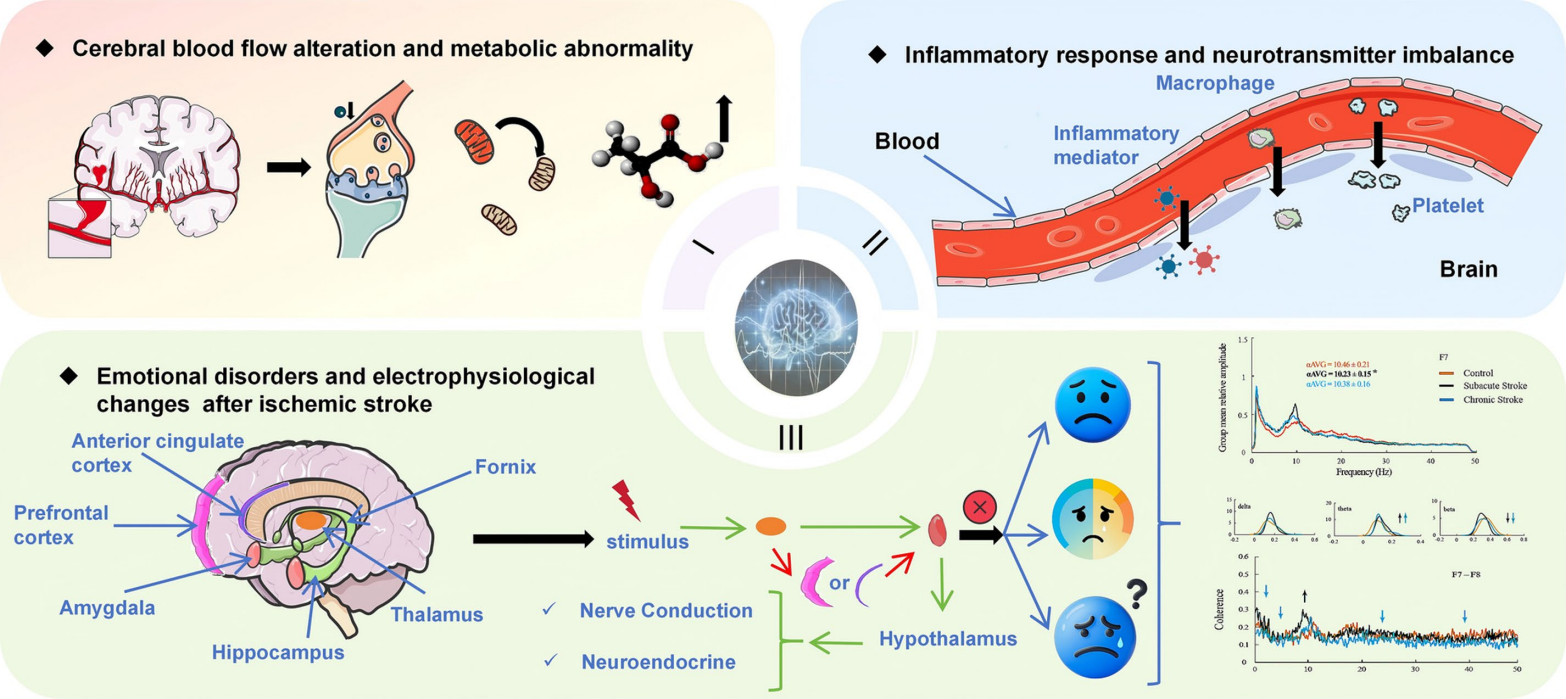
Pathophysiology and Emotional Disorders in Ischemic Stroke. It is divided into three parts: (1) Cerebral Blood Flow and Metabolic Abnormalities: Ischemic stroke leads to reduced cerebral blood flow, causing metabolic disturbances such as decreased ATP production and increased lactate accumulation. (2) Inflammation and Neurotransmitter Imbalance: Stroke activates immune cells, releasing inflammatory mediators that disrupt neurotransmitter balance, contributing to cognitive and emotional disorder. (3) Emotional Disorders and Electrophysiological Changes: Stroke affects neural conduction and endocrine function in specific brain regions, leading to emotional problems. Electrophysiological indicators, such as slower EEG alpha generation and synchronization, reflect functional differences in brain activity under various conditions. The EEG waveband data graph in the lower- right corner is reproduced from Petrovic et al. (32), CC-BY 4.0.

A comprehensive analysis of the literature shows that the mental-disorder-related parameters monitored by qEEG vary significantly at different time periods after stroke. In the initial 72 h period, EEG assessment may demonstrate instability and trigger abnormal electrical activities across the cerebral cortex. This early stage is characterized by a complex and evolving neural landscape, where the brain undergoes immediate responses to the insult. Potential influencing factors could encompass acute inflammation, disrupted neurotransmitter balance, and alterations in cerebral blood flow (49, 50). Recent findings in neuroscience, such as the research by Vecchio et al. (51) and Huang et al. (52), suggest that these factors interact in a complex manner to influence the qEEG parameters during this critical period. To visualize this process, we have prepared a figure, as shown in Figure 2.

From 72 h to 1 month after the stroke, distinct patterns such as the significant decrease in beta power within two weeks post-infarction and the relative power ratio of theta in the lesion ipsilateral occipital area emerged as predictive factors (24, 49). This indicates a transition in the neural processes and the establishment of certain stable patterns related to cognitive and functional outcomes. The reasons for these changes might involve neural plasticity, recovery of neural circuits, or compensatory mechanisms. Current research on neural repair and remodeling, such as the studies by Dhamoon et al. (53) and Bögli et al. (49), provides valuable context for understanding these changes.

Beyond 1 month post-stroke, slow wave activities, particularly in the affected hemisphere, demonstrated predictive value for functional recovery. This suggests that neural reorganization and adaptation persist over an extended period. The potential mechanisms could involve long-term remodeling of neural networks and changes in synaptic efficacy (53, 54). Insights from studies on chronic neural changes, such as the work by Song et al. (55) and Hu et al. (56), offer additional perspectives on these long-term processes.

The inconsistent changes in qEEG parameters over time highlight the necessity for longitudinal studies that can capture the dynamic nature of neural recovery and the development of mental disorders after a stroke. Such studies would enable a more comprehensive understanding of the temporal trajectory of these changes and facilitate the development of targeted therapeutic interventions based on specific time points. Future research should incorporate more cutting-edge and authoritative literature to deeply analyze the mechanisms and influencing factors of these changes, promote the development of this field, and improve the prognosis of patients.

### 4.1 Outlook

Quantitative EEG has garnered significant attention for predicting psychiatric disorders following ischemic stroke. However, several issues persist in current research. Primarily, studies have predominantly focused on the correlation between monitoring data at specific time points and mental disorders, neglecting the importance of continuous EEG monitoring. While some longitudinal assessments have been conducted, they have primarily involved correlation analyses of power spectra. Additionally, although qEEG has addressed the limitations of traditional EEG in terms of time consumption and expertise required, there are still numerous indicators that require monitoring. Previous studies have identified certain indicators such as power spectral density and coherence, but a comprehensive set of indicators is currently lacking. Therefore, it is essential to establish an objective method that facilitates early and accurate diagnosis, thereby assisting experts in reducing treatment time and aiding patients and their families in comprehending and supporting the disease process.

The complexity and varying severity of post-stroke mental disorders complicate their diagnosis in affected patients. Currently, there are no clear guidelines or consensus regarding the monitoring of this condition, resulting in the absence of a standardized diagnostic and treatment model. Establishing such a model would not only enhance the reliability and comparability of research findings but would also ultimately contribute to improving the quality of life for post-stroke patients. Future research should implement monitoring and evaluation in accordance with relatively recognized standardized diagnostic and treatment models. Additionally, it should compare and analyze a series of indicators related to qEEG, with the goal of identifying the most effective EEG characteristics for diagnosing mental disorders and establishing alarm thresholds for early intervention. Future research should concentrate on examining various subtypes of mental disorders and formulating personalized treatment strategies aimed at enhancing patient outcomes, minimizing complications, expediting recovery, improving quality of life, and ensuring the efficient utilization of resources. With the advancements in science and technology, the application of wearable technology and mobile health can enhance the effective monitoring of mental health in stroke patients, making it more accessible and convenient. This, in turn, contributes to the development of standardized and personalized diagnosis and treatment models.

### 4.2 Limitations of our systematic literature analysis

In this study, our intention was to conduct a comprehensive analysis of the existing literature on electroencephalogram monitoring at different time periods in ischemic stroke. Nevertheless, due to the significant heterogeneity in the research designs of the included studies, which encompasses variations in the characteristics of the study population, differences in intervention methods, and diversity in measurement indicators, performing a meta-analysis to synthesize the relevant results is not feasible. This heterogeneity poses challenges for directly comparing and integrating the results of individual studies. As a result, we did not pool the data and did not conduct a meta-analysis, and thus cannot provide a combined effect measure in this review.

## 5 Conclusion

This systematic review expounds on the application progress of qEEG in post-ischemic stroke mental disorders. The main findings are that specific qEEG features are associated with mental disorders, and these features vary across different time periods. For future research, it is essential to focus on specific types of post-stroke mood disorders for in-depth exploration. Additionally, multi-center and longitudinal studies should be carried out to improve the qEEG indicator system, aiming to standardize and enhance the monitoring of this disease.

## Data Availability

The original contributions presented in the study are included in the article/supplementary material, further inquiries can be directed to the corresponding author.
